# Lithium side effects and toxicity: prevalence and management strategies

**DOI:** 10.1186/s40345-016-0068-y

**Published:** 2016-12-17

**Authors:** Michael Gitlin

**Affiliations:** Geffen School of Medicine, University of California Los Angeles, Los Angeles, USA

**Keywords:** Lithium, Side effects, Renal function, Thyroid, Nephrotoxicity

## Abstract

Despite its virtually universal acceptance as the gold standard in treating bipolar disorder, prescription rates for lithium have been decreasing recently. Although this observation is multifactorial, one obvious potential contributor is the side effect and toxicity burden associated with lithium. Additionally, side effect concerns assuredly play some role in lithium nonadherence. This paper summarizes the knowledge base on side effects and toxicity and suggests optimal management of these problems. Thirst and excessive urination, nausea and diarrhea and tremor are rather common side effects that are typically no more than annoying even though they are rather prevalent. A simple set of management strategies that involve the timing of the lithium dose, minimizing lithium levels within the therapeutic range and, in some situations, the prescription of side effect antidotes will minimize the side effect burden for patients. In contrast, weight gain and cognitive impairment from lithium tend to be more distressing to patients, more difficult to manage and more likely to be associated with lithium nonadherence. Lithium has adverse effects on the kidneys, thyroid gland and parathyroid glands, necessitating monitoring of these organ functions through periodic blood tests. In most cases, lithium-associated renal effects are relatively mild. A small but measurable percentage of lithium-treated patients will show progressive renal impairment. Infrequently, lithium will need to be discontinued because of the progressive renal insufficiency. Lithium-induced hypothyroidism is relatively common but easily diagnosed and treated. Hyperparathyroidism from lithium is a relatively more recently recognized phenomenon.

## Background

Despite its place as the gold standard for maintenance treatment in bipolar disorder, prescription patterns from a number of (but not all) countries demonstrate a decreasing use of lithium (Karanti et al. 2016; Kessing et al. 2016; Parabiaghi et al. 2015). Assuredly, this phenomenon reflects a number of factors that influence both physician and patient behaviors including the number of other mood stabilizers available, the need for regular monitoring via venipuncture with lithium, the marketing of other patent-protected mood stabilizers and so forth. Beyond the decision as to which mood stabilizer should be prescribed, some of these same factors are likely to play a role in predicting adherence to maintenance lithium.

Side effects are another important variable in both prescription patterns and adherence. Surprisingly, the exact role of side effects in predicting lithium nonadherence—which averages >40% (Perlick et al. 2004)—is still unclear (Goodwin and Jamison 2007). Clinicians may view side effects as more important in nonadherence than do patients (Jamison et al. 1979). Additionally, patients’ perception of or apprehension of side effects, as opposed to the actual presence of side effects may contribute more to nonadherence (Scott and Pope 2002). Specific side effects—such as cognitive dulling—may also be more associated with nonadherence than the total number of side effects (Gitlin et al. 1989). Complicating the issue is the frequent misattribution of symptoms as side effects with a common example being cognitive dullness as a symptom of depression, attributed to the mood stabilizer, as a side effect. Nonetheless, it seems self-evident that side effects play at least some role in lithium nonadherence.

Because of this, knowledge of lithium side effects and education about these with patients remains an essential part of clinical practice. Additionally, managing these side effects remains a critical element in psychiatrists’ optimal treatment of bipolar disorder. This paper reviews the most common side effects of lithium and reviews treatment strategies for them. It also reviews the potential toxic effects of lithium on organ function since managing these risks is also essential in long-term lithium therapy.

## Methods

A literature review was conducted for papers through July 2016 using the online search engine PUBMED, with the key words “lithium” AND “side effects”. Additional searches were generated using “lithium” and the specific side effects described in the paper. This search was supplemented by cross referencing and by the use of classic texts (Goodwin and Jamison 2007; Bauer et al. 2006). The focus of this review is prevalence rates and clinical management strategies. Pathophysiological mechanisms are only included for a few side effects/toxicities (e.g., renal effects) for which it was felt to be relevant for education and management strategies.

## Results

### Overall side effect burden

In evaluating physical complaints from any specific patient, three factors should always be considered: (1) misattribution of symptoms for side effects; (2) the effect of the mood state itself—specifically depression—on subjective side effect burden (Gitlin et al. 1989; Wilting and Heerdink 2009). Bipolar patients who are currently depressed consistently endorse greater side effect burden than euthymic patients. Because of this, consideration should be given to more aggressive treatment of depression to alleviate some of the subjective side effect burden before considering dose changes or adding antidotes. (3) The last factor to consider is the additive effect of multiple pharmacotherapies on side effect rates. This is especially important given the frequency with which bipolar patients are treated with polypharmacy. As an example, in the STEP-BD study, the average bipolar patient was taking three medications (Goldberg et al. 2009). It is reasonable to assume that, for instance, weight gain, which is a common side effect with many medications prescribed for bipolar patients, can be additive across multiple agents. In these situations, the role of any one medication—e.g., lithium—in causing the side effect cannot be known. Non-prescribed medications should also be considered as potential additive factors in evaluating side effects. As examples, caffeinated beverages can add to the tremor caused by lithium while over-the-counter hypnotics such as antihistamines can increase sedation or fatigue.

In older studies, with data collected during a time when more (but certainly not all) patients were seemingly treated with lithium monotherapy, the majority of lithium-treated patients report at least one side effect with estimates ranging between 67 and 90% (Johnston et al. 1979; Bone et al. 1980; Vestergaard et al. 1980; Gitlin et al. 1989). Thus, only a relative small fraction of lithium-treated patients are side effect free. Most patients have more than one side effect attributed by the patient to lithium (Vestergaard et al. 1980; Gitlin et al. 1989).

In managing side effects from lithium, basic strategies are the same as for all medications, as listed in Table 1.

**Table 1:** Managing lithium side effects: overall strategies

Watchful waiting
Lower dose
Alter time of medication administration
Changing to a different lithium formulation
Antidotes for specific side effects
Change medication to a different mood stabilizer

The utility of these strategies will be discussed more specifically in the sections below describing individual side effects. Watchful waiting assumes that tolerance to that specific side effect occurs. Tolerance occurs with only some side effects, e.g., nausea but not weight gain. Similarly, dose adjustments may be helpful with some but not all side effects. Surprisingly, specific dose/side effect relationships are not well established for a number of side effects. The lithiumeter provides an example of considering the optimal balance between lithium efficacy and side effects (Malhi et al. 2011). Altering the time of the lithium ingestion should always be considered when managing some but not all side effects. Changing the lithium to a different preparation—from capsules to sustained release or vice versa—is only useful for side effects affected by absorption such as gastrointestinal side effects. Antidotes to specific side effects are only available inconsistently. The risk/benefit ratio of antidotes will differ according to the patient and the antidote. Some patients are uneasy about adding a second medication simply to treat the side effects of the first medication. Additionally, the side effects associated with the antidote must always be considered. Changing the mood stabilizer is, of course, the most drastic response to side effects and should be done only when the side effects are unmanageable or the associated subjective distress intolerable.

### Gastrointestinal side effects: nausea and diarrhea

Gastrointestinal side effects—typically nausea and/or diarrhea—are relatively common side effects from lithium and have been recognized since the earliest lithium studies. Nausea, seen in 10–20% of lithium-treated patients, tends to be more prominent early in treatment and is much less common in long-term therapy (Schou et al. 1970). Nausea is not among the most distressing of lithium side effects and is only infrequently associated with nonadherence or drug discontinuation. Because nausea may correlate with lithium levels, especially peak levels, taking lithium after meals, using a multiple daily dose regimen or using sustained release preparations may diminish nausea. Because tolerance to the nausea commonly occurs over time, these strategies can often be discontinued over time with the resumption of once-daily administration of lithium capsules. Vomiting is infrequent except in the setting of lithium toxicity and would then be accompanied by other side effects such as ataxia and the emergence of gross tremor.

Diarrhea increases in prevalence in patients through the first 6 months of treatment and is seen in up to 10% of lithium-treated patients (Vestergaard et al. 1988). Serum lithium levels >0.8 mEq/l are associated with higher rates of diarrhea (Vestergaard et al. 1988). Some, but not all studies, suggest higher rates of diarrhea with sustained release lithium preparations, presumably due to more distal absorption of the drug (Edstrom and Persson 1977). As with vomiting, lithium intoxication can be associated with diarrhea. The presence or absence of other toxic symptoms and a serum lithium level will help guide the clinician as to whether further evaluation for toxicity should be considered.

### Polyuria/polydipsia

Excessive urination and thirst (polyuria and polydipsia) are consistently found to be among the most common side effects associated with lithium with rates up to 70% in long-term patients (Bone et al. 1980; Vestergaard et al. 1980; Gitlin et al. 1989). However, patients generally rate these side effects as less annoying and less likely to precipitate lithium discontinuation compared to other side effects (Gitlin et al. 1989). Polyuria is typically (and arbitrarily) defined as >3000 ml/24 h. The presumption is that the thirst associated with lithium is secondary to the obligate renally mediated polyuria. The mechanism by which lithium causes polyuria is its interference with the collecting tubules to generate cyclic adenosine monophosphate in response to antidiuretic hormone stimulation. This results in a reduction of the kidneys’ capacities to preserve free water, leading to impaired concentrating ability and the production of excessively dilute urine (Gitlin 1999). This effect is initially functional, seen early in treatment and is reversible (like alcohol’s similar effect), later progressing to structural and irreversible changes. It is unclear whether early renal symptoms, such as polyuria, are predictive of later renal damage. (Renal damage from lithium is discussed below in the section on organ toxicity).

Proposed risk factors for the renal effects leading to polyuria/polydipsia include: duration of treatment, higher serum lithium levels, repeated episodes of lithium intoxication and the ingestion of other psychotropic medications, especially antipsychotics (Gitlin 1999; Azab et al. 2015).

Whether lithium dosage regimens, i.e., once-daily vs. divided dose, correlate with polyuria risk is still debated (Gitlin 1999; Carter et al. 2013). A number of naturalistic, observational studies suggested that once-daily lithium was associated with lower urine volume (=less subjective polyuria) (Schou et al. 1982; Bowen et al. 1991). This is consistent with animal data (Plenge et al. 1981). Once-daily lithium regimens are associated with higher peak levels but, maybe more importantly, lower trough levels. Low lithium levels may be needed for renal regenerative processes (Plenge et al. 1982). Many of the random assignment comparing different dosage regimens evaluated patients who were long-term lithium patients and who, therefore, may already have had structural, irreversible changes. In the only study randomizing newly treated lithium patients, once-daily dosing was associated with lower urinary frequency (Singh et al. 2011).

Treatments for dry mouth are purely symptomatic and unrelated to the cause. They include sugarless gum or glycerin-based oral moisturizers. Occasionally, cholinergic mouthwashes or oral preparations that contain pilocarpine may be helpful.

For polyuria, the optimal treatment is prevention—keeping lithium levels as low as feasible, avoiding toxicity episodes and once-daily lithium dosing. If the latter strategy is used, it should be implemented as early in treatment as possible before structural damage occurs. Paradoxically, diuretics are an established remedy for lithium-induced polyuria. Amiloride, typically dosed at 5 mg bid has been demonstrated as effective (Finch et al. 2003). Hydrochlorothiazide, usually at 50 mg daily is also effective (MacNeil et al. 1975). Since lithium levels increase with concomitant thiazide treatment, it is critical that the lithium dose is reduced by approximately 1/3 and then checked a few days later to avoid lithium toxicity. Additionally, since thiazides decrease potassium levels, the latter should be measured and potassium supplements may be required. Alternatively, combination preparations with a thiazide and a potassium sparing diuretic such as triamterene may be administered.

### Tremor

Tremor, primarily of the hands, is among the most common lithium side effects, seen in approximately one quarter of treated patients (Gelenberg and Jefferson 1995). Lithium tremor is generally symmetric and is indistinguishable from essential or physiologic tremor. Thus, it is most apparent with intentional posture, such as writing or holding a coffee cup (Baek et al. 2014). It is distinct from the parkinsonian tremor associated with dopamine-blocking agents. Patients treated with *D*2 blockers and lithium may present with a complex tremor from multiple etiologies. The severity of lithium-induced tremor is additive to other forms of physiologic tremor from etiologies such as anxiety, alcohol withdrawal, caffeine ingestion or idiopathic, familial tremor. Some studies have found additive effects from other medications such as some antidepressants (Bone et al. 1980; Vestergaard et al. 1988).

Lithium-induced tremor typically presents early in treatment but may emerge at any time. When it occurs later, additional etiologies should be considered such as those noted above. At times, lithium tremor seems to improve spontaneously after years of treatment. Lithium tremors are more common with older age, presumably due to the additive effects of age-related essential tremor. The type of lithium preparation does not alter tremor prevalence but higher lithium levels correlate with greater risk of tremor (Vestergaard et al. 1988).

Tremor is exceedingly common in the context of lithium toxicity. During lithium toxicity, the tremor tends to be coarser, more irregular, more widespread (affecting other body parts), more severe and is associated with the other symptoms of toxicity.

Most cases of lithium-induced tremor are managed conservatively by reducing or eliminating additive factors such as reducing caffeine and keeping lithium levels in the low–medium range. If the tremor is relatively mild, many patients simply live with it. When conservative measures are ineffective and the tremor is moderate in intensity or socially embarrassing, antidotes should be considered. The most common treatment is beta-blockers, as established by two small double-blind studies and multiple case reports, series and clinical experience (Baek et al. 2014). This is consistent with the larger literature on beta-blockers for essential tremor (Deuschl et al. 2011). Propranolol is the most commonly prescribed beta-blocker for lithium-induced tremor although other agents seem effective too. Typical propranolol doses for tremor range from 20 to 320 mg although most patients are effectively treated in daily doses <120 mg. Other agents that may be considered if beta-blockers are either ineffective or not well tolerated include primidone, benzodiazepines (especially if anxiety is a contributing factor to the tremor) and Vitamin B6 (Minodownik et al. 2002).

### Weight gain

Weight gain is among the prevalent and distressing of lithium-associated side effects. In one study, although it was the third most common side effect, patients rated it as the most bothersome and the second most bothersome side effect that might result in lithium discontinuation (Gitlin et al. 1989). Definitions of weight gain and duration of observation differ across studies, precluding any simple average of lithium-induced weight gain. However, all studies find weight gain in a substantial proportion of lithium-treated patients. Typical results include those of Vestergaard et al. (1980) who found that 20% of patients gained 10 kg or more. In another study, 77% of lithium-treated patients gained weight with an average increase of 6.3 kg (8% baseline body weight) (Chengappa et al. 2002). These results are remarkably similar to the 73% rate of weight gain in the Aarhus clinic (Vestergaard et al. 1988). Among more recent studies, mean weight change over one year in one double-blind study of lithium-treated patients was 4.2 kg (Calabrese et al. 2003). Weight gain may be maximal in the first 1–2 years of treatment in at least one study (Vestergaard et al. 1988). Baseline body weight predicts greater weight gain (Vendsborg et al. 1976; Vestergaard et al. 1988). Weight gain correlates with dose/lithium levels in some (Abou-Saleh and Coppen 1989; Gelenberg et al. 1989) but not all (Vestergaard et al. 1988) studies. Of course, for the majority of bipolar patients on other psychotropic medications other than lithium, weight gain should be considered as due to multiple agents such as antipsychotics, valproate and some antidepressants.

Mechanisms of lithium-associated weight gain are still unclear. Certainly, thirst leading to the ingestion of high-calorie drinks may play a role in some patients (Peselow et al. 1980). Unrecognized hypothyroidism and edema may play a role in a small minority of patients. However, it seems likely that, for most patients, lithium alters other core mechanisms that cause weight gain (Ackerman and Nolan 1998).

Suggested treatment strategies for weight gain are all based on common sense and nonspecific approaches. First, the likelihood of weight gain should be discussed before lithium treatment is initiated since prevention is easier than treatment. Patients should be encouraged to drink low or noncaloric drinks to treat their thirst. General diet and exercise strategies should, of course, be encouraged. If the patient is taking multiple medications, switching from a treatment with high weight gain liability (such as olanzapine or quetiapine) to another with less weight gain (e.g., aripiprazole or lurasidone) should be considered. Finally, if these strategies are insufficient, the use of adjunctive weight-losing medications, such as topiramate may be tried (Chengappa et al. 2001).

### Cognitive impairment

Negative effects on cognition from a medication are exceedingly distressing to patients. As with other potential side effects, however, patients’ attribution of the etiology of cognitive dysfunction and/or dullness may be incorrect. Cognitive dysfunction has been well described as being associated with bipolar disorder itself, evident while patients are euthymic (Bourne et al. 2013) or when depressed (Malhi et al. 2007). Associating lithium (correctly) with the diminution of manic/hypomanic periods with the loss of the unusual cognitive sharpness and creativity creates another confound. Additionally, distinguishing the effect of lithium from other psychotropic medications being prescribed—antipsychotics, serotonergic antidepressants, benzodiazepines—is often impossible. Nonetheless, bipolar patients list cognitive dysfunction, manifested by mental slowness, as the side effect most likely to precipitate lithium nonadherence (Gitlin et al. 1989).

Early studies described cognitive (and affective) dullness from lithium which showed a clear dose–response pattern (Schou 1968; Szmulewicz et al. 2016). Jamison, in her review of this topic in her classic text concluded that lithium does cause anterograde amnesia, slightly slowed motor movement and diminished creativity (Goodwin and Jamison 2007). In contrast, in a meta-analysis of the 12 studies published at that time, it was concluded that lithium had only minor negative effects on cognition and only in the domains of verbal learning and memory (effect size [ES] = 0.24 and creativity (ES = 0.33)) (Wingo et al. 2009). More recently, Malhi and colleagues have reviewed the effects of lithium on different neurocognitive domains and suggested optimal cognitive tests in patients for whom further testing is indicated (Malhi et al. 2016).

The decrease in creativity, best demonstrated by an on/off study of idiosyncratic associations, may be particularly troublesome to the subset of bipolar patients involved in creative professions, such as writing, music and art (Shaw et al. 1986). There are no suggested systematic treatment strategies for lithium-associated cognitive dysfunction. Clinically, a first consideration should be to lower the lithium serum level since cognitive effects seem dose related. Second, a review of other psychotropic medications being prescribed and whether they might contribute to the side effect would be in order. Finally, although never evaluated in any systematic manner, stimulants should be considered. Modafinil and armodafinil have demonstrated safety in bipolar disorder with no evidence of increasing the risk of affective switch (Frye et al. 2015). More controversial—but still worthy of consideration in selected cases—would be the use of dopaminergic stimulants such as methylphenidate or d-amphetamine to enhance cognitive function.

### Sexual function

In contrast to most of the other potential side effects surveyed in this paper, sexual dysfunction from lithium has been relatively neglected as a topic of clinical inquiry. A recent literature review found only thirteen papers on the topic (Elnazer et al. 2015). Problems in interpreting the little data that exist include a lack of control groups since sexual dysfunction in epidemiological samples is considerable (Laumann et al. 1999); the negative effect of other psychotropic medications (especially antidepressants and antipsychotics) on sexual function; the potential effect of depressed mood and depressive symptoms on sexuality. An earlier study suggested that sexual dysfunction in lithium-treated patients was seen only in patients also taking benzodiazepines (Ghadirian et al. 1992). In one of the few studies using a control group of age-matched healthy controls, stable bipolar patients on lithium showed decreased libido and sexual satisfaction (Zuncheddu and Carpiniello 2006). Both the onset of bipolar disorder and the institution of lithium seemed to have independent negative influences on sexuality. In the most recent study, 37% of euthymic bipolar patients on lithium acknowledged sexual dysfunction across multiple sexual domains (Grover et al. 2014).

Given the paucity of data in this area, unsurprisingly, few treatment approaches to lithium-associated sexual dysfunction have been suggested. In the only controlled trial, aspirin 240 mg daily was more effective than placebo in reducing overall sexual dysfunction and in improving erectile dysfunction (Saroukhani et al. 2013). Phosphodiesterase 5 inhibitors, which have been demonstrated as effective in treating SSRI-induced sexual dysfunction in both men and women (Nurnberg et al. 2008, 2003), should be considered for those with lithium-associated sexual difficulties, especially if diminished arousal plays an important role.

### Dermatological effects

Exact prevalence rates of dermatological disorders from lithium are not available. Both new cases and exacerbation of pre-existing acne and psoriasis due to lithium have been described (Pfennig et al. 2006) with one study suggesting a significantly higher risk in men (Chan et al. 2000). The latter finding may reflect the higher prevalence of acne in young men vs. young women, presumably due to the influence of testosterone. Moderate to severe psoriasis should be considered a relative contraindication to lithium. Dermatological effects of lithium may be related to lithium levels.

Thus, a first therapeutic strategy for lithium-associated acne would be to consider lowering the lithium dose. In mild cases of acne, usual dermatological remedies should be considered. A small placebo-controlled trial found a positive effect of inositol 6 g daily in decreasing the severity of psoriatic lesions in lithium-treated patients (Allen et al. 2004). However, if the skin lesions are moderate to severe, do not respond to conventional remedies and/or are associated with substantial social self-consciousness (especially in young people), switching from lithium to another mood stabilizer may be necessary.

### Lithium intoxication

As has been well known since the early days of its use in bipolar disorder, lithium has a narrow therapeutic index, with relatively little space between therapeutic and toxic levels. Because of this, avoidance of lithium intoxication has been and continues to be an important goal in treatment. Early reports suggested mortality rates from lithium toxicity ranging from 9 to 25% (Hansen and Amdisen 1978). However, recent data suggest mortality rates of much less than 1% (Baird-Gunning et al. 2016). As an example, in 2012 in the United States, only 11 deaths occurred from 6815 toxic exposures to lithium (Mowry et al. 2013) yielding a mortality rate of 0.16%. Lithium toxicity has been divided into three patterns: acute, acute-on-chronic and chronic with the two latter forms being more dangerous since they are associated with more time to distribute lithium to the CNS intracellular space. In mild lithium toxicity, symptoms include weakness, worsening tremor, mild ataxia, poor concentration and diarrhea. With worsening toxicity, vomiting, the development of a gross tremor, slurred speech, confusion and lethargy emerge (Bauer and Gitlin 2016). Etiologies of lithium intoxication include intentional or accidental overdose, and any factor that alters salt and/or water balance such as the initiation of new medications that alters lithium excretion, dehydration, and infections with fever (Hansen and Amdisen 1978; Haussmann et al. 2015; Ott et al. 2016). Given the potential consequences of lithium toxicity, particular care and vigilant monitoring should be core treatment components with older patients given lithium, since they are more vulnerable to lithium intoxication and at far lower levels than younger patients. Additionally, lithium-treated patients should be queried regularly about their potential use of other medications that may interfere with lithium excretion and, therefore, increase the likelihood of lithium toxicity such as ACE inhibitors, nonsteroidal anti-inflammatory medications such as diclofenac, indomethacin and COX-2 inhibitors such as celecoxib.

The most important potential irreversible sequelae from lithium intoxication are neurological, especially cerebellar dysfunction including ataxia, dysarthria and dysmetria (Munshi and Thampy 2005).

Treatment guidelines for lithium intoxication vary depending on the degree of toxicity. In cases of mild toxicity, lithium discontinuation may suffice. With moderate toxic episodes, fluid infusion with saline diuresis is recommended along with gastric lavage (if the intoxication is recognized early) and whole bowel irrigation using polyethylene glycol. In the most severe cases, defined by extraordinarily high lithium levels (>4.0 mmol/l) or marked clinical symptoms, especially altered consciousness, extracorporeal methods such as hemodialysis should be instituted. If hemodialysis is required, it is usually done repeatedly to avoid lithium rebound caused by a redistribution of lithium from deeper compartments or red blood cells to the plasma (Decker et al. 2015). More detailed treatment recommendations can be found elsewhere (Decker et al. 2015; Haussmann et al. 2015; Baird-Gunning et al. 2016).

### Long-term effects on organ systems

The three organ systems that may be negatively affected by lithium are the thyroid gland, kidneys and parathyroid glands.

## Lithium and the kidneys

The polyuria discussed above reflects lithium’s effect on the renal tubular system. Concerns that this might reflect structural irreversible damage, as opposed to simply reversibly interfering with tubular function, began with the first reports of biopsy-proven interstitial nephritis in lithium-treated patients almost 40 years ago (Hestbech et al. 1977). All studies examining renal morphology in lithium-treated patients have consistently found the same results: focal nephron atrophy, and interstitial fibrosis with relative preservation of glomeruli (Gitlin 1999). This is consistent with the clinical features of lithium-associated nephropathy—obligate polyuria—but without marked decrease in filtering capacity of the kidneys as measured by eGFR and secondarily by serum creatinine. (The latter measure is less accurate than eGFR since it also reflects muscle mass which decreases with age. Thus, an older person may have substantially diminished eGFR but a relatively normal serum creatinine.) Polyuria correlates only weakly with reduced kidney function with the former rather common and the latter unusual (Azab et al. 2015).

Although lithium-treated patients have, in general, a lower eGFR than those not treated, the eGFR does not correlate with time on lithium suggesting that it is not progressive within groups. However, a subgroup of lithium-treated patients does show progressive renal insufficiency. This is manifested by “creeping creatinine” (Jefferson 1989) with a gradual rise in serum creatinine and a decrease in creatinine clearance over years. This phenomenon occurs in approximately 20% of lithium-treated patients (Lepkifker et al. 2004). In one study, approximately 1/3 of lithium-treated patients had an eGFR <60 ml/min while 5% showed an eGFR of <30 ml/min (Aiff et al. 2015).

An even smaller subgroup of lithium-treated patients progresses towards end-stage renal disease (ESRD) and ultimately dialysis and/or renal transportation. The prevalence of ESRD associated with lithium is difficult to estimate. One study found the risk to be almost eightfold compared to the general population (Aiff et al. 2014a). In contrast, another study found risk for renal insufficiency but not ESRD (Kessing et al. 2015) while a third study found no differences in the rate of eGFR declines in lithium-treated patients vs. those treated with other psychotropic agents (Clos et al. 2015). In the most recent study, compared to patients treated with other mood stabilizers such as valproate, olanzapine or quetiapine, lithium was associated with higher rates of chronic renal disease (eGFR <60 ml/min) but not more severe renal disease (eGFR <30 ml/min) (Hayes et al. 2016). Since lithium-associated ESRD is virtually exclusively seen in patients treated for a very long term—in one study, the average time on lithium for those with ESRD was 27 years (Aiff et al. 2014a), studies of only 10–15 years may not show the increase in ESRD.

Some recent studies have suggested both lower rates of ESRD in lithium-treated patients over the last thirty years when mean therapeutic lithium levels are lower than before (Aiff et al. 2014b) and less effect on renal function in general with lower levels (Aprahamian et al. 2014). However, the largest study of unselected patients continued to find significant rates of renal damage and ESRD in a lithium-treated population (Aiff et al. 2015).

Risk factors for lithium-induced nephropathy are length of treatment, age, and prior episodes of lithium toxicity (Aiff et al. 2015; Bocchetta et al. 2015; Clos et al. 2015). Whether the lithium regimen—once-daily vs. multiple doses—predicts differential rates of ESRD is unclear.

Some evidence exists that progressive renal impairment continues even after lithium discontinuation, (Bocchetta et al. 2015). In one study, patients with a serum creatinine >2.5 mg/dl (220 ml/l) at the time of lithium discontinuation were far more likely to progress to ESRD (Bendz et al. 2010). In another study, further deterioration of renal function was more likely if the creatinine clearance was <40 ml/min (Presne et al. 2003).

General guidelines for minimizing the risk of significant renal damage in lithium-treated patients are: monitor serum creatinine and eGFR regularly during lithium treatment at intervals of every 6 months to 1 year; avoid episodes of lithium toxicity, keep mean lithium levels within the low therapeutic range when possible, and consider once-daily dosing. When the serum creatinine rises to 1.6 mg/dl (140 mmol/l), consultation with a nephrologist is appropriate and consideration for lithium discontinuation should be discussed with the patient (Gitlin 1993). Since the progression of renal damage is slow, if discontinuing lithium is deemed necessary, the second mood stabilizer should be added, titrated to full dose and only then should the lithium be tapered and discontinued gradually over 4–8 weeks.

### Lithium and thyroid function

First recognized in the late 1960s when goiters were discovered in a cohort of lithium-treated patients (Schou et al. 1968), antithyroid effects of lithium are now well established. Multiple mechanisms are probably involved. The most important of these is inhibition of thyroid hormone release from the thyroid gland; however, lithium may also decrease iodine trapping with the gland and inhibit synthesis of thyroid hormones (Lazarus 2009; Kibrige et al. 2013).

The prevalence of thyroid dysfunction in lithium-treated patients varies substantially across studies, reflecting both different populations and varying definitions of hypothyroidism. As examples, lithium has been associated with overt hypothyroidism (manifest symptoms of hypothyroidism plus high thyroid-stimulating hormone [TSH] and low T4) vs. subclinical hypothyroidism (asymptomatic plus high TSH with normal T4) vs. goiter without reference to biochemical markers. Overt hypothyroidism is estimated as having a prevalence of 8–19% with subclinical hypothyroidism showing rates up to 23% (Kleiner et al. 1999). Many patients with goiter are euthyroid in that the enlarged gland has been sufficiently stimulated to synthesize and release adequate amounts of thyroid hormone.

The most important risk factors for lithium-induced hypothyroidism are the presence of antithyroid antibodies, which increases the risk by eightfold (Bocchetta et al. 2007), female sex (which covaries with antithyroid antibody prevalence (Ozerdem et al. 2014; Kraszewska et al. 2015; Shine et al. 2015), older age and a family history of hypothyroidism (Kibrige et al. 2013).

Symptoms of lithium-induced hypothyroidism are the same as seen in primary cases of the disorder—lethargy, mental slowing, depression, weight gain, dry skin, and cold intolerance. Of course, a number of these symptoms overlap with depression symptoms as well as side effects from lithium or other psychotropic agents, making diagnosis difficult in the absence of thyroid function tests.

Given the substantial rates of thyroid dysfunction in lithium-treated patients, thyroid parameters should be checked before lithium is instituted and then monitored after 3–6 months initially and then every 6–12 months. The minimal test required both before and during lithium treatment is the thyroid-stimulating hormone (TSH) level. Peripheral thyroid hormone measurements T3 and T4 are recommended by some, as are antithyroid (TPO) antibodies and/or ultrasound of the thyroid gland.

The interpretation and recommendations for clinical management of thyroid abnormalities during lithium treatment varies. The most important clinical rule is that hypothyroidism never justifies lithium discontinuation. A reasonable middle ground approach of Kleiner et al. (1999) suggests that TSH values >10 mU/L on two occasions should be interpreted as incipient thyroid gland failure and warrants administration of l-thyroxine even if the patient is asymptomatic. TSH levels between top normal and slightly high (usually 4–4.5 and 10 mU/L) should be treated if the patient has refractory depression or lassitude/fatigue. TSH levels between 4 and 10 mU/L in asymptomatic patients can be monitored closely without exogenous thyroid treatment, although some experts add l-thyroxine in these situations also. Thyroid hormones should be prescribed to bring TSH values within the normal range.

Although not systematically studied, patients with lithium-induced hypothyroidism who discontinue lithium can usually stop thyroid treatment. Occasionally, however, discontinuing l-thyroxine after lithium discontinuation results in the re-emergence of hypothyroidism. In these cases, it is assumed that lithium exacerbated a subclinical hypothyroidism which then continued after lithium discontinuation.

### Lithium and parathyroid gland/calcium

Although evidence of increased calcium and serum parathyroid hormone (PTH) associated with lithium treatment has been available for decades, only recently has this effect been examined more systematically. Lithium increases renal calcium reabsorption and independently stimulates parathyroid hormone release (Shapiro and Davis 2015). A meta-analysis of relevant studies found that lithium treatment was associated with a 10% increase in blood calcium and PTH levels (McKnight et al. 2012). Studies published since then have mostly confirmed this finding (Albert et al. 2013, 2015; Shine et al. 2015). There is some evidence that lithium-associated hypercalcemia/hyperparathyroidism is associated with fewer clinical symptoms than are seen in primary hyperparathyroidism (Shapiro and Davis 2015). Classic symptoms of hypercalcemia include weakness, fatigue, renal stones, renal insufficiency and osteoporosis. Because of how recent these findings about lithium and calcium levels are, most Practice Guidelines with some exceptions (Yatham et al. 2013; Malhi et al. 2015) do not yet recommend regular monitoring of calcium and PTH levels with lithium-treated patients. However, measuring calcium levels both before the initiation of lithium treatment and yearly during treatment would seem to be prudent.

Treatment of lithium-associated hypercalcemia/hyperparathyroidism is the same as for primary cases (Shapiro and Davis 2015). Mild elevations of levels in asymptomatic patients can be simply monitored. With higher levels, switching from lithium to a different mood stabilizer, calcimimetic therapy with cinacalcet or local or subtotal parathyroidectomy are the reasonable treatment options.

## Conclusion

Side effects and potential toxicities underlie at least part of the decreased utilization of lithium over the last decade or more. A number of these side effects—polyuria, thirst, nausea, tremor, sexual side effects—are typically either mild or no worse than annoying. Others such as weight gain and cognitive dullness/impairment are more distressing to patients and may be more associated with nonadherence. However, of note, in direct comparison studies, dropout rates due to other than relapse do not differ between lithium and anticonvulsant comparators (Severus et al. 2014). A number of basic nonspecific strategies, summarized in Table 1, may suffice for managing these side effects. In other circumstances, specific remedies, summarized in Table 2, may be implemented to diminish patients’ distress and to enhance the likelihood of treatment adherence.

**Table 2:** Managing lithium side effects: treatment strategies

Side effect	Treatment strategies
Polyuria	Once-daily dosing diuretics
Thirst/polydipsia	Sugarless gum glycerin-based oral moisturizers cholinergic mouthwashes
Tremor	Beta-blockers primidone benzodiazepines Vitamin B6
Weight gain	Avoid high-calorie drinks exercise/diet topiramate
Cognitive impairment	Stimulants
Sexual dysfunction	Aspirin phosphodiesterase 5 inhibitors
Skin lesion—acne–psoriasis	Usual remedies, inositol

In most cases, lithium toxicity is preventable. Proper education and monitoring will certainly diminish the number of toxic episodes in lithium-treated patients.

Potential organ toxicity requires more vigilance both because of the need for laboratory monitoring of TSH, serum creatinine and eGFR and calcium levels and the potential consequence of these toxicities if and when they emerge. With proper monitoring, these concerns can be easily managed in the vast majority of lithium-treated patients. The small but measurable increased risk for ESRD in lithium-treated patients cannot be prevented completely but with the use of lower therapeutic lithium levels, monitoring of eGFR and judicious discontinuation of lithium when needed, this risk can be minimized and patients more effectively treated.

## References

[CR1] Abou-Saleh MT, Coppen A (1989). The efficacy of low-dose lithium: clinical, psychological and biological correlates. J Psychiatr Res.

[CR2] Ackerman S, Nolan LJ (1998). Body weight gain induced by psychotropic drugs: incidence, mechanisms and management. CNS Drugs..

[CR3] Munshi KR, Thampy A (2005). The syndrome of irreversible lithium-effectuated neurotoxicity. Clin Neuropharmacol..

[CR4] Aiff H, Attma P, Aurell M, Bendz H, Schon S, Svedlund J (2014). End-stage renal disease associated with prophylactic lithium treatment. Eur Neuropsychopharmacol.

[CR5] Aiff H, Attman P, Aurell M, Bendz H, Schon S, Svedlund J (2014). The impact of modern treatment principles may have eliminated lithium-induced renal failure. J Psychopharmacol..

[CR6] Aiff H, Attman P, Aurell M, Bendz H, Ramsauer B, Schon S, Svedlund J (2015). Effects of 10–30 years of lithium treatment on kidney function. J Psychopharmacol..

[CR7] Albert U, De Cori D, Aguglia A, Barbaro F, Lanfranco F, Boggetto F (2013). Lithium-associated hyperparathyroidism and hypercalcaemia: a case-control cross-sectional study. J Affect Disord.

[CR8] Albert U, De Cori D, Aguglia A, Barbaro F, Lanfranco F, Bogetto F (2015). Effects of maintenance lithium treatment on serum parathyroid hormone and calcium levels: a retrospective longitudinal naturalistic study. Neuropsychiatr Dis Treat..

[CR9] Allen SJR, Kavanagh GM, Herd RM, Savin JR (2004). The effect of inositol supplements on the psoriasis of patients taking lithium: a randomized, placebo-controlled trail. B J Dermatol..

[CR10] Aprahamian I, Santos FS, dos Santos B, Talib L, Diniz BS, Radanovic M (2014). Long-term, low- dose lithium treatment does not impair renal function in the elderly: a 2-year randomized, placebo-controlled trial followed by single blind extension. J Clin Psych..

[CR11] Azab AN, Shnaider A, Osher Y, Wang D, Bersudsky Y, Belmaker RH (2015). Lithium nephrotoxicity. Int J. Bipolar Disord..

[CR12] Baek JH, Kinrys G, Nierenberg AA (2014). Lithium tremor revisited: pathophysiology and treatment. Acta Pyschiatr Scand..

[CR14] Baird-Gunning J, Lea-Henry T, Hoegberg LCG, Gosselin S, Roberts DM (2016). Lithium poisoning. J Intensive Care Med..

[CR15] Bauer M, Grof P, Muller-Oberlinhausen B (2006). Lithium in neuropsychiatry: the comprehensive guide.

[CR16] Bauer M, Gitlin M (2016). The essential guide to lithium treatment.

[CR17] Bendz H, Schon S, Attman P, Aurell M (2010). Renal failure occurs in chronic lithium treatment but is uncommon. Kidney Int.

[CR18] Bocchetta A, Cocco F, Velluzzi F, Del Zompo M, Mariotti S, Loviselli A (2007). Fifteen-year follow up of thyroid function in lithium patients. J Endocrinol Invest.

[CR19] Bocchetta A, Ardau R, Fanni T, Sardu C, Piras D, Pani A (2015). Renal function during long-term lithium treatment: a cross-sectional and longitudinal study. BMC Med.

[CR20] Bone S, Roose SP, Dunner DL, Fieve RR (1980). Incidence of side effects in patients on long-term lithium therapy. Am J Psychiatry.

[CR21] Bourne C, Aydemir O, Balanza-Martinez V, Bora E, Brissos S, Cavanagh JT (2013). Neuropsychological testing of cognitive impairment in euthymic bipolar disorder: an individual patient data meta-analysis. Acta Psychiatr Scand.

[CR22] Bowen RC, Grof P, Grof E (1991). Less frequent lithium administration and lower urine volume. Am J Psychiatry.

[CR23] Calabrese JR, Bowden CL, Sachs G (2003). A placebo-controlled 18-month trial of lamotrigine and lithium maintenance treatment in recently depressed patients with bipolar I disorder. J Clin Psychiatry.

[CR24] Carter L, Zolezzi M, Lewczyk A (2013). An updated review of the optimal lithium dosage regimen for renal protection. Can J Psychiatry.

[CR25] Chan HHL, Wing Y, Su R, Van Krevel C, Lee S (2000). A control study of the cutaneous side effects of chronic lithium therapy. J Affect Disord.

[CR26] Chengappa KNR, Levin J, Rathore D (2001). Long-term effects of topiramate on bipolar mood instability weight change and glycemic control: a case-series. Eur Psychiatry..

[CR27] Chengappa KN, Chalasani L, Brar JS (2002). Changes in body weight and body mass index among psychiatric patients receiving lithium, valproate, or topiramate: an open-label, non-randomized chart review. Clin Ther.

[CR28] Clos S, Rauchhaus P, Severn A, Cochrane L, Donnan PT (2015). Long-term effect of lithium maintenance therapy on estimated glomerular filtration rate in patients with affective disorders: a population-based cohort study. Lancet.

[CR29] Decker BS, Goldfarb DS, Dargan PI, Friesen M, Gosselin S, Hoffman RS (2015). Extracorporeal treatment for lithium poisoning: systematic review and recommendations from the EXTRIP work group. Clin J Am Soc Nephrol..

[CR30] Deuschl G, Raethjen J, Hellriegel H, Elble R (2011). Treatment of patients with essential tremor. Lancet Neurol.

[CR31] Edstrom A, Persson G (1977). Comparison of side and lithium sulphate preparations giving medium-slow and slow-release. Acta Psychiatr Scand.

[CR32] Elnazer HY, Sampson A, Baldwin D (2015). Lithium and sexual dysfunction: an under-researched area. Hum Psychopharmacol Clin Exp..

[CR33] Finch CK, Kelley KW, Williams RB (2003). Treatment of lithium-induced diabetes insipidus with amiloride. Pharmacotherapy..

[CR34] Frye MA, Amchin J, Bauer M, Adler C, Yang R, Ketter TA (2015). Randomized, placebo-controlled, adjunctive study of armodafinil for bipolar I depression: implications of novel drug design and heterogeneity of concurrent bipolar maintenance treatments. Int J Bipolar Disorder..

[CR35] Gelenberg AJ, Kane JM, Keller MB (1989). Comparison of standard and low serum levels of lithium for maintenance treatment of bipolar disorder. N Engl J Med.

[CR36] Gelenberg AJ, Jefferson JW (1995). Lithium tremor. J Clin Psychiatry.

[CR37] Gitlin M (1993). Lithium-induced renal insufficiency. J Clin Psychopharmacol.

[CR38] Gitlin M (1999). Lithium and the Kidney. An updated review. Drug Safety..

[CR39] Gitlin M, Cochran SD, Jamison KR (1989). Maintenance lithium treatment: side effects and compliance. J Clin Psychiatry.

[CR40] Ghadirian AM, Annable L, Belanger MC (1992). Lithium, benzodiazepines, and sexual function in bipolar patients. Am J Psychiatry.

[CR41] Goldberg JF, Brooks JO, Kurita K, Hoblyn JC, Ghaemi SN, Perlis RH (2009). Depressive illness burden associated with complex polypharmacy in patients with bipolar disorder: findings from the STEP-BD. J Clin Psychiatry.

[CR42] Goodwin FK, Jamison KR (2007). Manic-depressive illness: Bipolar disorders and recurrent depression.

[CR43] Grover S, Ghosh A, Sarkar S, Chakrabarti S, Avasthi A (2014). Sexual dysfunction in clinically stable patients with bipolar disorder receiving lithium. J Clin Psychopharmacol.

[CR44] Hansen HE, Amdisen A (1978). Lithium intoxication (Report of 23 cases and review of 100 cases from the literature). Q J Med..

[CR45] Haussmann R, Bauer M, von Bonin S, Grof P, Lewitzka U (2015). Treatment of lithium intoxication: facing the need for evidence. Int J Bipolar Disord..

[CR46] Hayes JF, Marston L, Walters K, Geddes JR, King M, Osborn DP (2016). Adverse renal, endocrine, hepatic and metabolic events during maintenance mood stabilizer treatment for bipolar disorder: a population-based cohort study. PLoS ONE.

[CR47] Hestbech J, Hanson HE, Amdisen A, Olsen S (1977). Chronic renal lesions following long-term treatment with lithium. Kidney Int.

[CR48] Jamison KR, Gerner RH, Goodwin FK (1979). Patient and physician attitudes towards lithium: relationship to compliance. Arch Gen Psychiatry.

[CR49] Jefferson JW (1989). Lithium: a therapeutic magic wand. J Clin Psych..

[CR50] Johnston BB, Dick EG, Naylor GJ, Dick DAT (1979). Lithium side effects in a routine lithium clinic. Br J Psych..

[CR51] Karanti A, Kardell M, Lundberg U, Landen M (2016). Changes in mood stabilizer prescription patterns in bipolar disorder. J Affect Disord.

[CR52] Kessing LV, Gerds TA, Feldt-Rasmussen B, Anderson PK, Licht RW (2015). Use of lithium and anticonvulsants and the rate of chronic kidney disease. A nationwide population-based study. JAMA..

[CR53] Kessing LV, Vradi E, Anderson PK (2016). Nationwide and population-based prescription patterns in bipolar disorder. Bipolar Disord.

[CR54] Kibrige D, Luzinda K, Ssekitoleko R (2013). Spectrum of lithium induced thyroid abnormalities: a current perspective. Thyroid Res..

[CR55] Kleiner J, Altshuler L, Hendrick V, Hersham JM (1999). Lithium-induced subclinical hypothyroidism: review of the literature and guidelines for treatment. J Clin Psychiatry.

[CR56] Kraszewska A, Chlopocka-Wozniak M, Abramowicz M, Sowinski J, Rybakowski JK (2015). A cross-sectional study of thyroid function in 66 patients with bipolar disorder receiving lithium for 10–44 years. Bipolar Disord.

[CR57] Laumann EO, Paik A, Rosen RC (1999). Sexual dysfunction in the united states: prevalence and predictors. JAMA.

[CR58] Lazarus JH (2009). Lithium and thyroid. Best Pract Res Clin Endocrinol Matab..

[CR59] Lepkifker E, Sverdlik A, Iancu I, Ziv R, Segev S, Kotler M (2004). Renal insufficiency in long-term lithium treatment. J Clin Psychiatry.

[CR60] McKnight RF, Adida M, Budge K, Stockton S, Goodwin GM, Geddes JR (2012). Lithium toxicity profile: a systematic review and meta-analysis. Lancet.

[CR61] MacNeil S, Hanson-Nortey E, Paschalis C, Eastwood PR, Jenner FA (1975). Diuretic during lithium therapy. Lancet.

[CR62] Malhi GS, Ivanovski B, Hadzi-Pavlovic D, Mitchell PB, Vieta E, Sachdev P (2007). Neuropsychological deficits and functional impairment in bipolar depression, hypomania and euthymia. Bipolar Disord.

[CR63] Malhi GS, Tanious M, Gershon S (2011). The lithiumeter: a measured approach. Bipolar Disord.

[CR64] Malhi GS, Bassett D, Boyce P, Bryant R, Fitzgerald PB, Fritz K (2015). Royal Australian and New Zealand college of psychiatrists clinical practice guidelines for mood disorders. Aust N Z J Psychiatry.

[CR65] Malhi GS, McAulay C, Gershon S, Gessier D, Fritz K, Das P, Outhred T (2016). The lithium battery: assessing the neurocognitive profile of lithium in bipolar disorder. Bipolar Disorder..

[CR66] Minodownik C, Witztum E, Lerner V (2002). Lithium-induced tremor treated with vitamin b6: a preliminary case series. Int J Psychiatry Med.

[CR67] Mowry JB, Spyker DA, Cantilena LR, Bailey JE, Ford M (2013). 2012 Annual report of the American association of poison control centers’ national poison data system (NPDS): 30th annual report. Clin Toxicol..

[CR68] Nurnberg HG, Hensley PL, Gelenberg AJ, Fava M, Lauriello J, Paine S (2003). Treatment of antidepressant-associated sexual dysfunction and sildenafil: a randomized controlled trail. JAMA.

[CR69] Nurnberg HG, Hensley PL, Heiman JR, Croft HA, Debattista C, Paine S (2008). Sildenafil treatment of women with antidepressant-associated sexual dysfunction: a randomized controlled trail. JAMA.

[CR70] Ott M, Stegmayr B, Salander Renberg E, Werneke U (2016). Lithium intoxication: incidence, clinical course and renal function—a population-based retrospective cohort study. J Psychopharacol..

[CR71] Ozerdem A, Tunca Z, Cimrin D, Hidirouglu C, Ergor G (2014). Female vulnerability for thyroid function abnormality in bipolar disorder: role of lithium treatment. Bipolar Disord.

[CR72] Parabiaghi A, Barbato A, Risso P, Fortino I, Bortolotti A, Merlino L (2015). Lithium use from 2000 to 2010 in Italy: a population-based study. Pharmacopsychiatry..

[CR73] Perlick DA, Rosen RA, Kaczynski R (2004). Medication nonadherence in bipolar disorder: a patient-centered review of research findings. Clin Approaches Bipolar Disord..

[CR74] Peselow ED, Dunner DL, Fieve RR, Lautin A (1980). Lithium carbonate and weight gain. J Affect Disord.

[CR75] Pfennig A, Deshauer D, Albrecht G (2006). Dermatologic effect of lithium: adverse reactions and potential therapeutic utility—Lithium in Neuropsychiatry: the comprehensive guide.

[CR76] Plenge P, Mellerup ET, Norgaard T (1981). Functional and structural rat kidney changes caused by peroral or parenteral lithium treatment. Acta Psychiatr Scand.

[CR77] Plenge P, Mellerup ET, Bolwig TG, Brun C, Hetmar O, Ladegoged J (1982). Lithium treatment: does the kidney prefer one daily does instead of two?. Acta Psychiatr Scan..

[CR78] Presne C, Fakhouri F, Noël LH, Stengel B, Even C, Kreis H, Mignon F, Grünfeld JP (2003). Lithium-induced nephropathy: rate of progression and prognostic factors. Kidney Int.

[CR79] Saroukhani S, Emami-Parsa M, Modabbernia A, Ashrafi M, Farokhnia M, Hajiaghaee R, Akhondzadeh S (2013). Aspirin for treatment of lithium-associated sexual dysfunction in men: randomized double-blind placebo-controlled study. Bipolar Disord.

[CR80] Schou M (1968). Lithium in psychiatric therapy and prophylaxis. J Psychiatr Res.

[CR81] Schou M, Amdisen A, Jensen S, Olston T (1968). Occurrence of goiter during lithium treatment. Br Med J.

[CR82] Schou M, Baastrup PC, Grof P, Weis P, Angst J (1970). Pharmacological and clinical problems of lithium prophylaxis. Br J Psychiatry.

[CR83] Schou M, Amdisen A, Thomsen K, Vestergaard P, Hetmar O, Et Mellerup (1982). Lithium treatment regimen and renal water handling: the significance of dosage pattern and tablet type examined through comparison of results from two clinics with different treatment regimens. Psychopharmacology.

[CR84] Scott J, Pope M (2002). Nonadherence with mood stabilizers: prevalence and predictors. J Clin Psychiatry.

[CR85] Severus E, Taylor MJ, Sauer C, Pfenning A, Ritter P, Bauer M, Geddes JR (2014). Lithium for prevention of mood episodes in bipolar disorders: systematic review and meta-analysis. Int J of Bipolar Disorders..

[CR86] Shapiro HI, Davis KA (2015). Hypercalcemia and “primary” hyperparathyroidism during lithium therapy. Am J Psychiatry.

[CR87] Shaw ED, Mann JJ, Stokes PE, Manevitz AZ (1986). Effects of lithium carbonate on associative productivity and idiosyncrasy in bipolar outpatients. Am J Psych..

[CR88] Shine B, McKnight RF, Leaver L, Geddes JR (2015). Long-term effects of lithium on renal, thyroid, and parathyroid function: a retrospective analysis of laboratory data. Lancet.

[CR89] Singh LK, Nizamie SH, Akhtar S, Praharaj SK (2011). Improving tolerability of lithium with a once-daily dosing schedule. Am J Ther.

[CR90] Szmulewicz A, Samame C, Caravotta P, Martino DJ, Igoa A, Hidalgo-Mazzei D (2016). Behavioral and emotional adverse events of drugs frequently use in the treatment of bipolar disorders: clinical and theoretical implications. Int J Bipolar Disord..

[CR91] Vendsborg PB, Bech P, Rafaelsen OJ (1976). Lithium treatment and weight gain. Acta Psychiatr Scan..

[CR92] Vestergaard P, Amdisen A, Schou M (1980). Clincally significant side effects of lithium treatment. Acta Psychiatr Scand.

[CR93] Vestergaard P, Poulstrup M, Schou M (1988). Prospective studies on a lithium cohort 3. Tremor, weight gain, diarrhea, psychological complaints. Acta Psychiatr Scand.

[CR94] Wilting I, Heerdink ER, Nolen WA, Egberts ACF, den Boer JA, Mersch PPA (2009). Association between lithium serum level, mood state, and patient-reported adverse drug reactions during long-term lithium treatment: a naturalistic follow-up study. Bipolar Disord.

[CR95] Wingo AP, Wingo TS, Harvey PD, Baldessarini RJ (2009). Effects of lithium on cognitive performance: a meta-analysis. J Clin Psychiatry.

[CR96] Yatham LN, Kennedy SH, Parikh SV, Schaffer A, Beaulieu S, Alda M (2013). Canadian Network for mood and anxiety treatments (CANMAT) and international society for bipolar disorders (ISBD) collaborative update of CANMAT guidelines for the management of patients with bipolar disorder: updated 2013. Bipolar Disord.

[CR97] Zuncheddu C, Carpiniello B (2006). Sexual dysfunctions and bipolar disorder: a study of patients submitted to a long-term lithium treatment. Clin Ther..

